# Protein energy wasting; what is it and what can we do to prevent it?

**DOI:** 10.1007/s00467-019-04424-2

**Published:** 2019-12-13

**Authors:** Lesley Rees

**Affiliations:** Department of Paediatric Nephrology, Great Ormond Street Hospital for Children NHS Foundation Trust, WC1N 3JH, London, UK

**Keywords:** Protein energy wasting, Chronic kidney disease, Cachexia

## Abstract

Some children with declining height and BMI SDS fail to respond to optimisation of nutritional intake. As well as poor growth, they have muscle wasting and relative preservation of body fat. This is termed protein energy wasting (PEW). The process results from an interaction of chronic inflammation alongside poor nutritional intake. This review discusses the causes and potential preventative therapies for PEW.

## Introduction

Ensuring that the nutritional intake of children with chronic kidney disease (CKD) is adequate and appropriate has been repeatedly demonstrated to improve growth, particularly in the very young. However, even in those who are receiving a calorie and protein intake that is judged to be optimal, catch-up growth may be incomplete. This is especially the case for children on dialysis [1].

It would be expected that children who are malnourished due to inadequate intake would maintain their appetite and respond promptly to provision of protein and energy. Such children adapt to their nutritional deprivation by decreasing their energy expenditure and metabolising body fat in preference to muscle mass and protein stores. However, in some children with chronic disease factors other than inadequate intake are contributing to poor growth because only a partial response is seen after the provision of increased nutrients. These children have a maladaptive response to their poor growth as they maintain a high resting energy expenditure, loose lean body mass and maintain body fat mass so that the relative proportion of fat to lean body mass may even be increased. Appetite is often suppressed. This has been termed protein energy wasting (PEW). Table 1 shows the differences between malnutrition and PEW. PEW is especially important because of its association not only with poor growth but also with hospitalisation, cardiovascular disease and mortality [2].

**Table 1:** Differences between malnutrition and protein energy wasting (PEW)

Malnutrition	Protein energy wasting
Appetite increased	Appetite decreased
Inadequate intake of nutrients	Inadequate intake of nutrients only partially responsible
Low resting energy expenditure	High resting energy expenditure
Body fat is lost	Normal or increased fat mass
Lean body mass initially preserved, later loss of muscle mass and protein stores	Loss of lean body mass so relative increase in the proportion of body fat
Can be reversed by dietary supplements	Inadequate response to dietary supplements

## How do we define PEW in children with CKD?

The term PEW was proposed by the “International Society of Renal Nutrition and Metabolism” (ISRNM) (www.RenalNutrition.org) and defined in 2008 as metabolic and nutritional derangements characterised by decreased body stores of protein and energy (body protein and fat masses) [3]. The diagnosis was further characterised in adults to include in addition low serum albumin, low cholesterol and decreased protein intake [4]. Cachexia is a term used as an alternative to PEW. The ISRNM has suggested that cachexia should be reserved for severe PEW. However, definitions are similar, the main difference being that a loss of body weight > 5% is a mandatory criterion for cachexia but supportive for PEW [2].

A uniform definition for PEW is important, not just for defining management but also for comparing interventions and determining outcomes. Unfortunately, as yet, there are no internationally agreed criteria for making this diagnosis in children. In the growing patient, it is logical to include length velocity in the diagnostic criteria. The value of length velocity was confirmed in a study of 528 children from the CKiD cohort, with GFRs ranging from 30 to 90 mL/min/1.73 m^2^, which compared the addition of short stature or poor growth to the adult criteria for the definition of PEW. There was a better correlation with hospitalisation risk over 2 years when growth was included in the criteria for definition than when adult criteria alone were used [5]. The authors suggested that length velocity is more important than weight criteria in defining PEW in the paediatric population. CKiD has accepted this advice and adapted adult criteria for the diagnosis of PEW so that they are applicable to children. They suggest a combination of nutritional, growth and biochemical parameters, including reporting of decreased appetite; BMI or mid upper arm circumference for height age (the age at which the height would be on the 50th centile) of < 5th centile or a decline of ≥ 10% over a year; height < 3rd centile for height age or a fall in percentile ≥ 10% over a year; serum albumin < 3.8 g/L; cholesterol < 100 mg/dL; and transferrin < 140 mg/dL ([6].

## How do we determine whether a child has PEW (Table 2)?

Table 2 suggests some ways of evaluating the presence of PEW using simple criteria. Nutritional and growth assessments are a routine part of the management of all children with CKD, undertaken in all paediatric nephrology units, and are the most helpful in assessing the presence of nutritional problems, although not distinguishing between poor intake and PEW. Mid upper arm circumference measurement is less easy to perform and is not part of the nutritional assessment recommendations from KDOQI because of the levels of high intra- and inter-observer error [7]. A low serum albumin correlated well with PEW in the CKiD study, but it is well recognised that albumin can vary with volume status and can be unreliable in the situation of fluid overload on dialysis. On the other hand, low albumin is associated with inflammation as well as fluid overload, and both are associated with PEW and, in turn, with mortality. Other easy to measure criteria that are used in adults such as low transferrin or cholesterol were found to be very rare in the CKiD predialysis group and therefore not helpful, although they may be more so in children on dialysis [5]. They are part of the CKiD recommendations for the diagnosis of PEW. CRP may be helpful when looking for inflammation, but is not currently a diagnostic criterion for PEW in the CKiD definition [6].

**Table 2:** Suggested criteria for assessing PEW in children with CKD

Criterion	Evaluation
Dietary intake	Poor appetite
	Protein and energy less than recommended for age
Growth	Height < 3rd centile or declining height velocity despite optimal nutrition
	BMI below 5th centile or declining
	Mid upper arm muscle circumference < 5th centile or declining
Biochemical	Albumin below normal
	CRP high

Other methods of assessing body composition are mainly used for research rather than day to day clinical practise. Bioelectric impedance analysis and bioimpedance spectroscopy have both been proposed as means of determining lean body mass and fat mass [8] but are inaccurate when there is fluid overload. Dual energy X-ray absorptiometry (DEXA) has also been used in research situations and has been used to determine whole body and regional lean and fat mass in CKD [9].

## What are the consequences of PEW?

PEW in adults is associated with increased hospitalisation, CVD and mortality [10], and there is some evidence that this is the case in children too. Hypoalbuminaemia in children is associated with increased risk of hospitalisation. Of 416 US children receiving dialysis therapy, there was a U-shaped association between serum albumin and hospitalisation frequency: the hospitalisation rate was higher, at 2.7 per patient year in those with serum albumins < 3.5 or > 4.5 g/dL than those with in-between ranges, when the incidence was 1–6.1.9 per patient year [5]. Although the relationship with hypoalbuminaemia can be explained by fluid overload, inflammation, poor nutrition and/or PEW, the reason for the correlation with high levels is less clear but may be due to dehydration [11].

There is also an association between hypoalbuminaemia and mortality. Of 1949 patients in US Renal Data System, for each SD decrease in HtSDS, there was a 14% increase in risk for death, and for each SD decrease in growth velocity, the risk for death increased by 12%. Again, there was a U-shaped association between BMI and death [12]. While low BMI is likely to represent malnutrition or PEW, high BMI may be due to equally adverse features such as fluid overload and oedema or obesity. A further more recent study of 13,172 children again using USRDS confirmed that risk of death was higher in those who were underweight or overweight [13]. They also identified that risk of cardiac or infection-related death was higher in children with short stature at the time of first RRT [14].

CKiD has also developed a ‘frailty’ index for the paediatric population. ‘Frailty’ was originally developed in the elderly as a term to describe a vulnerable group of patients with a predisposition to complications and death. It is now used in many health conditions such as cancer and chronic diseases, including CKD. Many of the criteria for the CKiD frailty index overlap with their criteria for PEW, the difference being removal of biochemical parameters and addition of symptomatic fatigue and a CRP > 3 mg/dL. Children who had three of the criteria used for the CKiD definition of ‘frailty’ index had a threefold increased risk of infection and/or hospitalisation [15].

## How common is PEW in children?

The lack of a universal definition means that estimates of incidence will inevitably vary. Most large registries collect data on height and weight but do not distinguish between inadequate nutritional intake and PEW. However, what we do know is that muscle deficits become more common as CKD advances. A study using DEXA in 143 children with CKD identified that leg muscle mass deficits did not differ from controls in CKD 2 to 3, but were significantly and progressively lower than controls in CKD 4 to 5 and particularly so in those on dialysis, indicating increasing skeletal muscle wasting. Fat mass was increased compared with controls [9]. A study of 528 children from CKiD using their criteria for PEW (described above) found an incidence of 15% in these predialysis children [5]. In a further study of 557 children from CKiD, many had features of ‘frailty’ and PEW, with a prevalence of 39% suboptimal growth, 62% low muscle mass, 29% fatigue and 18% had a raised CRP [15].

## What are the causes of PEW? (Table 3)

Causes can be broadly broken down into two: inadequate nutrition and the effects of chronic inflammation.

**Table 3:** Causes of PEW

Nutrition	Inflammation
Poor appetite	The cause of the CKD
Abnormal taste	↑cytokine imbalance as CKD progresses
Preference for salt and water	Dialysis related infection
Multiple medications	CVL, exit site and peritonitis
Inadequate dialysis	Dialysis related inflammation
Leptin/ghrelin imbalance	Water impurity
Vomiting	Bio-incompatibility of dialyser and lines
Gastro-oesophageal reflux	↓response to anabolic hormones GH and IGF-1
Delayed clearance of polypeptide hormones affecting gastrointestinal motility	Fluid overload → poor tissue perfusion and absorption of gut toxins
Raised intra-abdominal pressure during PD	Metabolic acidosis
Loss of nutrients in dialysate	Oxidative stress
Fluid restriction	Abnormal gut microbiota
Fasting for medical procedures	Retention of uraemic molecules
Acute intercurrent illnesses	Retention of indoxyl sulphate and p-cresyl sulphate
	Post-transplant
	Use of steroids
	The failing graft
	Vitamin D deficiency

### Nutrition

#### Poor appetite

CKD affects taste sensation, which is reduced early in CKD and worsens as CKD progresses. The predominance of CAKUT (commonly a salt loosing condition) as a diagnosis means that such children have a preference for water and salty foods. Prescription of multiple medications may affect appetite. A high proportion of children, particularly in the very young, have co-morbid conditions which themselves influence intake (and growth) [16].

Disturbances of leptin and ghrelin have been implicated in control of appetite and the pathogenesis of PEW. Leptin, ghrelin and orbistatin are regulators of appetite and satiety: leptin is an appetite depressant and ghrelin and orbistatin appetite stimulants. They are degraded by the kidney so it has been postulated that an imbalance of these hormones might contribute to the disturbed appetite and PEW in CKD. However, results of levels in CKD are variable and inconsistent and their role remains unclear [17–19].

Inadequate dialysis may affect appetite [10]. Intensified dialysis is associated with improvement in nutritional status and catch-up growth [20].

#### Vomiting

Vomiting is common, particularly in the very young. It may result from gastro-oesophageal reflux and delayed gastric emptying, and can result in a significant loss of nutrients. Decreased clearance of polypeptide hormones may affect gastrointestinal motility, as does raised intra-abdominal pressure during PD [16].

#### Other nutritional causes

The need for a fluid restriction may limit nutritional intake in those who are dependent on a liquid diet. There are dialysate losses of protein in PD and amino acids in HD. Insufficient dietary intake will also occur during episodes of fasting surrounding surgical procedures and decreased intake during episodes of sepsis [16].

### Chronic inflammation

A state of chronic inflammation can be related both to the cause of the CKD and to CKD itself. As with all complications of CKD, there is worsening as CKD progresses.

#### Abnormalities of cytokines

Disturbances of cytokines occur with advancing CKD. Pro-inflammatory cytokines IL-1, IL-6 and TNF-α have been implicated in muscle breakdown. It is likely that individual cytokines play a role in particular pathological processes involved in PEW, such as anorexia and depression, resting energy expenditure and responsiveness to growth hormone and insulin-like growth factor 1 [21].

#### Dialysis

Infections are common in patients on dialysis. In HD, the presence of a central venous catheter increases infection risk; and during PD, exit site infections and peritonitis will contribute to inflammation. Components of the haemodialyser, lines and water that is not ultrapure may cause an inflammatory response [22]. Fluid overload also contributes by its effect on tissue perfusion and gut oedema with the subsequent absorption of bacterial toxins.

#### Decreased response to anabolic hormones

**Fig. 1:**
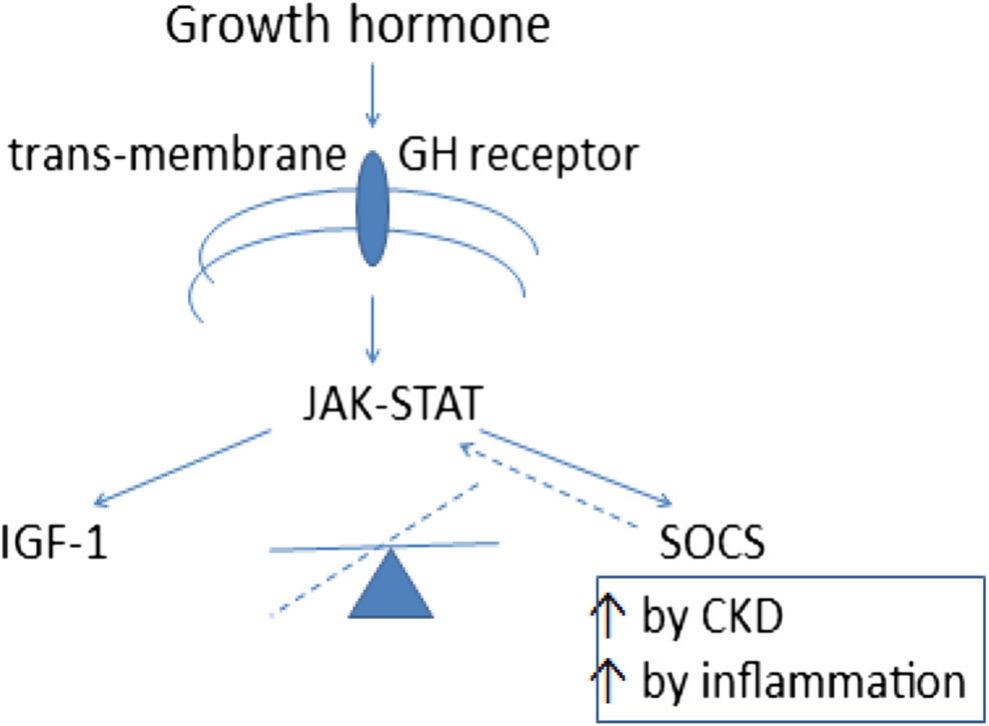
Effect of inflammation on growth hormone secretion. Reproduced from reference [23]

Inflammation contributes to the resistance to GH and IGF-1 that is an important factor contributing to the poor muscle bulk and growth in CKD (Fig. 1). GH binds to its receptor, resulting in tyrosine phosphorylation, activation of the JAK-STAT cascade and transcription of IGF-I synthesis and proteins of the suppressor of cytokine signalling (SOCS) family. SOCS dephosphorylates the GH-activated cascade and so exerts a GH-regulated negative feedback loop. In CKD, the balance between GH-induced transcriptional activation of IGF-I and SOCS is shifted towards SOCS overstimulation. As SOCS is induced by inflammatory cytokines, the chronic inflammatory state associated with CKD may contribute to SOCS overexpression and therefore GH resistance [19]. Decreased GH secretion is reported in malnutrition, metabolic acidosis and with steroid therapy, and IGF-1 bioactivity is decreased both due to deceased synthesis and also because of the accumulation of inhibitors in plasma (IGFBPs) [23].

#### Metabolic acidosis

The acidic environment may contribute to systemic inflammation by increasing cytokine production, particularly TNF-α; and β2-microglobulin production is increased. Acidosis is associated with breakdown of muscle, reduced albumin synthesis, insulin and GH resistance and poor growth [24].

#### Oxidative stress

Oxidative stress, an imbalance between increased pro-oxidant and deficient anti-oxidant capacity, is already present in early CKD. It causes inflammation via formation of pro-inflammatory oxidised lipids and advanced glycation end-products. In turn, nuclear factor κB (NFκB) transcription factor is activated, promoting the expression of pro-inflammatory cytokines such as TNF-α as well as recruiting leukocytes and other pro-inflammatory cells [25].

#### Change in gut microbiota, fluid overload and uraemic toxins

**Fig. 2:**
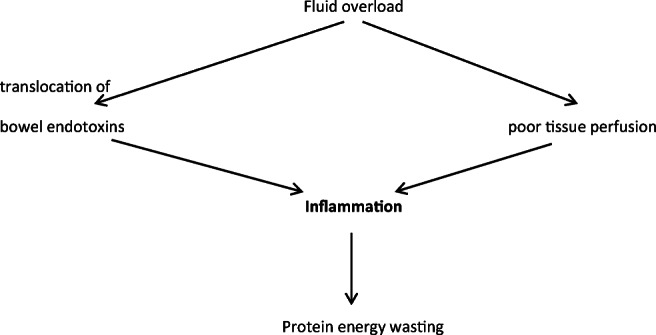
The role of fluid overload in the development of PEW

The interaction between the gut flora and health is a topic of emerging interest. In CKD, the gut microbiota may be altered by diet, which is often low in fibre in CKD, abnormal gut transit times, frequent use of antibiotics and other medications and acidosis. The gut microbiota becomes more pathogenic and disrupts the epithelial barrier in the gut wall. This and oedema due to fluid overload allow absorption of bacterial endotoxins, which trigger an immune response and inflammation (Fig. 2). Uraemic retention molecules, which come from endogenous metabolism, intestinal microbial metabolism or from diet, contribute to the inflammation.

The colon is responsible for final degradation of carbohydrates and proteins that have escaped absorption. This is achieved by fermentation. Indoxyl sulphate and p-cresyl sulphate are toxic by-products of this process that would normally be excreted by the kidneys. Their measurement can be used as an assessment of dialysis adequacy, as they represent removal of large molecular toxic metabolites [26, 27].

#### Other factors

##### Post-transplant

Steroids cause muscle wasting and also suppression of pituitary GH secretion. Another source of chronic inflammation is a failed kidney transplant in the patient on dialysis [28].

##### Vitamin D

Vitamin D has been shown to have anti-inflammatory effects. Low levels are very common and correlate with malnutrition but it is not clear whether this is cause or consequence. There is evidence in children on dialysis that low 1, 25(OH) (2)D levels are associated with inflammation: in 61 on dialysis, lower levels were associated with higher high-sensitivity C-reactive protein levels. High PTH levels may also contribute to the inflammatory process [29].

## What can we do to prevent PEW? (Table 4)

### Optimise nutrition

Optimal nutrition is of benefit in both malnutrition and PEW. KDOQI and the Paediatric Renal Nutrition Taskforce (PRNT) guidelines are an international reference standards defining optimum intake of protein/energy and all nutrients, how and how often to assess of growth parameters and how to prescribe and deliver a nutritional prescription. The early use of enteral tube feeding, as soon as growth is seen to be falling way from centiles, is recommended [7, 30]. Evidence for the benefit of enteral tube feeds on growth is extensively described in the very young, but also has been shown to benefit the nutritional state of children of all ages [31]. Both KDOQI and PRNT emphasise the importance of input from specialist renal paediatric dieticians.

**Table 4:** Ways to ameliorate PEW

Optimise nutrition	Regular dietary assessment
	Feed supplements
	Enteral tube feeding
Correct acidosis	Oral bicarbonate supplementation
	Adequate dialysis
Optimise dialysis	Prevention of infection
	Use of fistulae
	Intensified dialysis/HDF
	Water purification
Medication	RhGH
	Lowest possible steroid dosing
	Vitamin D
Exercise	Structured programmes
New treatments on the horizon	Appetite stimulants, manipulation of gut microbiome, anti-oxidant and anti-inflammatory medications

### Normalise serum bicarbonate

Treatment of metabolic acidosis by the provision of base to patients with CKD decreases the rate of protein degradation and urea generation, resulting in improved protein balance, increased muscle mass and growth [24]. Treatment can be by the oral route; KDOQI recommends maintaining the serum bicarbonate ≥ 22 mmol/L [7].

### Optimise dialysis

Inadequate clearance due to poorly functioning access during dialysis, or infectious or thrombotic complications of it, is a source of chronic inflammation. The better form of access is an AV fistula (AVF) in preference to a central venous line as AVF is associated with less infection, longer access survival and less hospitalisation [22].

Haemodiafiltration (HDF) seems to offer some benefit in reducing inflammation and oxidative stress and allows improved removal of middle molecules by convection. Of 22 children who were receiving conventional HD, biomarkers of inflammation (ß2-microglobulin, IL-6, IL-10, hCRP, oxidative stress (nitrotyrosine, advanced glycation end-products [AGEs], oxidised low-density lipoprotein [ox-LDL] and anti-oxidant capacity)) were increased even after 3 months of HD. However, after 3 months of HDF, there was a significant reduction in ß2-microglobulin, hCRP, AGEs and ox-LDL and an increase in total anti-oxidant capacity [32]. In a small study of 15 children undergoing daily HDF, nutritional status and growth and serum C-reactive protein (CRP) levels improved [20]. Home haemodialysis also enables more intensive dialysis and is associated with better growth and nutrition [22]. Water that is not ultrapure is another source of chronic inflammation on dialysis, and particularly so when large volumes are used, as in HDF [22]. Ensuring adequate fluid removal will also reduce inflammation and absorption of uraemic toxins [22].

### Medications

#### rhGH

Supra-physiological levels of GH are able to overcome the GH and IGF-1 resistance in CKD, with improvement in growth [23]. Growth hormone therapy also reduces muscle wasting; it is associated with greater leg lean mass SDS when adjusted for CKD severity [9].

#### Vitamin D

Given the well-recognised incidence of low vitamin D levels in CKD, and the association of low levels with inflammation, it is logical to ensure that patients are vitamin D–replete [33].

#### Steroids

Steroids may be part of the CKD therapy or part of post-transplant immunosuppression. Catch-up growth post-transplant is usually achieved in those on steroid-free regimen. The use of the minimal possible dose of steroids when they are unavoidable is therefore logical [23].

#### Appetite stimulants

Megestrol acetate, a synthetic progesterone derivative, has been shown to improve BMI SDS in 25 children over a period of up to 8 months and despite well-described side effects, was well tolerated. It may be a short-term strategy to improve nutritional status in children with CKD. Cyproheptadine has not yet been trialled in CKD [2].

### Exercise

Adults with CKD develop symptoms of fatigue and signs of muscle wasting leading to reduced exercise capacity and muscle weakness. Exercise programmes have been shown to improve these issues. Although research in children has not been able to demonstrate a positive outcome from attempts at increasing exercise, there is no reason to think that appropriately structured programmes in children would not result in similar benefits [34].

### New treatments

The role of dietary manipulations including fibre and probiotics to manipulate the gut microbiome; appetite simulants; and anti-inflammatory and anti-oxidant medications have been trialled but as yet are of unproven benefit [35].

## Conclusion

Protein energy wasting is a response to poor nutrition and chronic inflammation, characterised by decreased appetite, loss of muscle rather than fat mass and declining growth rate and BMI. Optimising nutrition is an important factor in treatment but may not completely reverse the abnormalities. It must be accompanied by reduction of sources of inflammation such as metabolic acidosis, suboptimal dialysis and oxidative stress.

## Multiple choice questions: (answers below the references)

1. Malnutrition differs from PEW because (one is true):

- Malnutrition is more common in younger children
- Appetite is lost in malnutrition but not PEW
- Body fat is preserved in malnutrition
- Loss of lean body mass occurs late in PEW
- Resting energy expenditure is high in PEW

2. Children with PEW have one of the following:

- Decreased fat mass
- Normal height velocity
- Reduced upper mid arm circumference
- Normal serum albumin
- Normal CRP

3. PEW is associated with one of the following:

- Muscle wasting but normal height velocity
- Increased risk of CVD
- No increased mortality risk
- Hospitalisation rate not increased
- Infection rate not increased

4. All but one of the following contributes to poor nutrition in PEW:

- Abnormal taste sensation
- high leptin levels
- Inadequate dialysis
- Gastro-oesophageal reflux
- Dialysate losses of fatty acids and triglycerides

5. All but one of the following contributes to inflammation in PEW:

- Decreased response to anabolic hormones
- Metabolic alkalosis
- Oxidative stress
- Fluid overload
- Increased levels of inflammatory cytokines as CKD progresses
